# Systematic Review on the Efficacy, Effectiveness, Safety, and Immunogenicity of Monkeypox Vaccine

**DOI:** 10.3390/vaccines11111708

**Published:** 2023-11-10

**Authors:** Ramy Mohamed Ghazy, Ehab Elrewany, Assem Gebreal, Rony ElMakhzangy, Noha Fadl, Eman Hassan Elbanna, Mahmoud M. Tolba, Elsayed Mohamed Hammad, Naglaa Youssef, Hazem Abosheaishaa, Elsayed Eldeeb Mehana Hamouda, Zeyad Elsayed Eldeeb Mehana, Ahmed Saad Al Zomia, Raad Ahmed A Alnami, Emad Ali Saeed Salma, Abdulaziz Saleh Alqahtani, Abdulaziz Fayez Alshehri, Mai Hussein

**Affiliations:** 1 Tropical Health Department, High Institute of Public Health, Alexandria University, Alexandria 21561, Egypt; ramy_ghazy@alexu.edu.eg (R.M.G.); ehabelrewany@alexu.edu.eg (E.E.); 2 Alexandria Faculty of Medicine, Alexandria University, Alexandria 21561, Egypt; assem.ahmed1801@alexmed.edu.eg (A.G.); hmad41337@gmail.com (E.M.H.); zeyadhamouda@gmail.com (Z.E.E.M.); 3 Family Health Department, High Institute of Public Health, Alexandria University, Alexandria 21561, Egypt; ronyibrahim13@hotmail.com (R.E.); nohaosama@alexu.edu.eg (N.F.); 4 Health Administration and Behavioral Sciences Department, High Institute of Public Health, Alexandria University, Alexandria 21561, Egypt; eman.elbanna@alexu.edu.eg; 5 Pharmaceutical Division, Ministry of Health and Population, Faiyum City 63723, Egypt; mahmoud.tolba@alumni2014.guc.edu.eg; 6 Medical-Surgical Nursing, Faculty of Nursing, Cairo University, Cairo 11562, Egypt; youssef_naglaa@cu.edu.eg; 7 Icahn School of Medicine at Mount Sinai, Elmhurst, NY 11373, USA; hazemabosheaishaa@gmail.com; 8 Department of Pathology, Faculty of Veterinary Medicine, Alexandria University, Alexandria 21561, Egypt; elsayedhamouda@gmail.com; 9 College of Medicine, King Khalid University, Abha 61421, Saudi Arabia; ahmedszomia@gmail.com (A.S.A.Z.); araad7768@gmail.com (R.A.A.A.); emad2009-1@hotmail.com (E.A.S.S.); abdulazizsalehaq@gmail.com (A.S.A.); abdulazizalshehri086@gmail.com (A.F.A.); 10 Clinical Research Administration, Alexandria Directorate of Health Affairs, Alexandria 21561, Egypt; 11 Egyptian Ministry of Health and Population, Cairo 11562, Egypt; 12 Master of Medical Science in Clinical Investigation, Harvard Medical School, Boston, MA 02115, USA

**Keywords:** monkeypox, vaccine safety, vaccine immunogenicity, vaccine effectiveness, vaccine efficacy

## Abstract

Background: The variation in the reported vaccine safety and effectiveness could contribute to the high rates of vaccine hesitancy among the general population and healthcare workers in areas where monkeypox (mpox) is circulating. In this review, our objective was to evaluate the safety, immunogenicity, effectiveness, and efficacy of the mpox vaccines. Methods: An extensive search for articles across multiple databases was performed, including searching six databases (PubMed Central, PubMed Medline, Scopus, Web of Science, Cochrane, ProQuest), two pre-print databases (European PMC Preprint and MedRxiv), and Google Scholar. Results: A total of 4290 citations were retrieved from the included databases. Following the removal of duplicates and the initial screening of records, a total of 36 studies were included into the analysis. Additionally, we identified five more studies through manual searches, resulting in a total of 41 eligible articles for qualitative synthesis. The study findings revealed that mpox vaccines demonstrate the ability to generate adequate antibodies; however, their effectiveness may decrease over time, exhibiting varying safety profiles. Most of the included studies consistently reported substantial levels of effectiveness and efficacy against mpox. Interestingly, the number of vaccine doses administered was found to influence the degree of immunogenicity, subsequently impacting the overall effectiveness and efficacy of the vaccines. Furthermore, we found that smallpox vaccines exhibited a form of cross-protection against mpox. Conclusions: Vaccines can be used to prevent mpox and effectively control its spread.

## 1. Introduction

Monkeypox virus (MPXV) is a DNA virus in the Poxvirida family, posing a significant threat to public health [1]. The World Health Organization (WHO) recognized the escalating monkeypox (mpox) outbreak as a Public Health Emergency of International Concern (PHEIC) on 23 July 2022. MPXV is prevalent in multiple countries across West and Central Africa [2]. Since 1 January 2022, WHO has received reports of mpox cases from 111 Member States spanning all six WHO regions. As of 23 May 2023, WHO has recorded a total of 1098 suspected cases and 87,529 laboratory-confirmed cases. These cases have resulted in 141 deaths [3]. Fortunately, the International Health Regulations (IHR) Emergency Committee on the multi-country outbreak of mpox held its fifth meeting on 10 May 2023. It concluded that this outbreak is no longer a PHEIC and gave updated interim guidance for a temporary period leading to a long-term mpox control strategy [4].

The MPXV is characterized by two separate lineages; namely, the Western African clade and the Central African clade. In general, the western African strain tends to cause less severe disease compared to the central African strain, with a lower case fatality rate (CFR) of 1–5% versus 10% [5]. In the current outbreak, the overall CFR was found to be 8.7%. It was 10.6% (95% CI of 8.4–13.3%) and 3.6% (95% confidence 1.7% to 6.8%) for the central and western African strains, respectively [6]. Until recently, sporadic and isolated cases of MPXV outside of the West and Central African regions were primarily linked to individuals who had recently traveled to areas where the MPXV is endemic [7]. The WHO Member States that are not considered endemic for virus have also reported cases of mpox. This transmission is concerning as it suggests a potential local transmission or a change in the pattern of MPXV spread [8]. Human transmission of infections often occurs through contact with infected animals or other humans. Once infected, large respiratory droplets or contact with a skin lesion can spread the virus from person to person, as well as indirect transmission through contaminated objects (fomites) [9]. The incubation period for the virus varies and can range between 7 and 21 days. The duration of the incubation period may be influenced by the nature of the exposure [10]. Individuals infected with mpox typically present with a self-limited clinical picture, characterized by a febrile prodrome, lymphadenopathy, and the development of vesicular-popular lesions [11,12].

Children and individuals with immunosuppressive conditions, including Human Immunodeficiency Virus (HIV) infection, are at a higher risk of experiencing severe disease. Encephalitis, secondary cutaneous bacterial infections, sepsis, dehydration, conjunctivitis, keratitis, and pneumonia are all common complications in endemic settings [13,14,15]. The secondary attack rate of mpox among unvaccinated household contacts reaches 10%. This means that there is a chance for transmission to occur among individuals who have not received the vaccine and reside in the same household as an infected person [16].

Given the quick global expansion of mpox, the accessibility of potent vaccines, and the ambiguous risk-benefit profile of the available antivirals, vaccination is anticipated to be a key factor in reducing mpox’s negative effects on the world [17]. It worth noting that the smallpox virus and mpox virus share a significant degree of genetic similarity. Historically, smallpox vaccines have proven to be effective in preventing or reducing the severity of mpox [18]. Via T cell epitope mapping, it has been determined that MPXV clade 1 and MPXV-2022 share at least 71% of the vaccinia virus (VACV) T cell epitopes, indicating possible cross-reactivity [19].

By immunizing individuals against mpox, we can reduce the transmission of the virus, mitigate outbreaks, and minimize the severity of the disease [20]. At present, mass vaccination for mpox is neither required nor recommended, considering the assessed risks and benefits, regardless of vaccine supply. Post-exposure prophylaxis (PEP) is recommended for individuals who have had contact with mpox cases. In order to avoid the onset of the disease, it entails giving a suitable second- or third-generation vaccination within 4 days of the initial exposure (up to 14 days in the absence of symptoms). Healthcare professionals (HCWs) at high risk of exposure, lab personnel dealing with Orthopoxvirus (OPXV), and clinical laboratory personnel engaged in diagnostic testing for mpox are all advised to use pre-exposure prophylaxis (PrEP) [18].

In 1980, Japanese regulatory authorities granted complete authorization for the live-attenuated vaccination LC16m8 [21]. As of 31 August 2007, the ACAM2000 vaccination received authorization from the Food and Drug Administration (FDA) for use in individuals at high risk of contracting smallpox [22]. This vaccine contains a replication-competent virus that can spread from the injection site to other parts of the body, and potentially to other individuals. Clinical studies have indicated that myopericarditis occurred at a rate of 5730 cases per million among recipients of ACAM2000. Progressive vaccinia, eczema vaccinatum, visual impairment, blindness, encephalopathy, encephalitis, encephalomyelitis, and other adverse effects have also been associated with this vaccine. Additionally, there have been reports of unvaccinated individuals dying after coming into contact with vaccinated individuals [23,24]. According to the Centers for Disease Prevention and Control (CDC) mpox and smallpox vaccines guidance, which was updated on 2 June 2022, a separate vaccine—called JYNNEOS, also known as Imvamune and Imvanex—is currently licensed in the United States of America (USA) for the prevention of smallpox. In 2019, the FDA approved JYNNEOS for smallpox and mpox prevention in adults at high risk of MPXV infection. Unlike ACAM2000, JYNNEOS is a live, non-replicating vaccine based on the Modified Vaccinia Ankara (MVA) strain. It is considered safer for use in immunocompromised individuals as it does not result in live virus production in vaccinated patients [25]. Multiple safety studies have been conducted on JYNNEOS. Participants with conditions such as HIV or atopic dermatitis, as well as previously immunized and unimmunized healthy adults, were involved in these studies. The results showed that these individuals did not experience significant adverse events or systemic reactions compared to those who received replicating vaccines [26]. In a noteworthy development, on 3 November 2021, the Advisory Committee on Immunization Practices (ACIP) decided to substitute ACAM2000 with JYNNEOS for the immunization of individuals who are at risk of orthopoxviral infection [27].

The variation in the reported vaccine safety and effectiveness could indeed contribute to the high rates of vaccine hesitancy among the general population and HCWs in areas where mpox is endemic [28,29]. When there are conflicting or inconsistent reports regarding the safety and effectiveness of vaccines, it can create uncertainty and doubt among individuals considering vaccination [30].

In this review, our objective was to evaluate the safety, immunogenicity, effectiveness, and efficacy of the existing mpox vaccines, including JYNNEOS, ACAM2000, and the live-attenuated LC16m8 vaccines. The findings of this review will offer invaluable insights for healthcare policymakers, enabling them to make well-informed decisions aimed at mitigating the morbidity and financial burden associated with mpox. Additionally, these insights will aid in proactively preventing potential outbreaks in regions where the virus continues to circulate.

## 2. Materials and Methods

### 2.1. Data Source and Search Strategy

The Preferred Reporting Items for Systematic Reviews and Meta-Analyses (PRISMA) standards were followed in the systematic review. The research team performed an extensive search for articles across multiple databases, including searching 6 databases (PubMed Central, PubMed Medline, Scopus, Web of Science, Cochrane, ProQuest), 2 preprint databases (European PMC Preprint and MedRxiv), and Google Scholar. The search encompassed all available timeframes and had no restrictions on geography or language. The search period extended up to 26 May 2023. The initial search was conducted by two authors, E.E. and A.G., and to ensure the search strategy’s accuracy and reliability, RMG independently revised the database search and verified the number of identified citations. The team also cross-checked the citations exported to the reference manager for consistency and completeness. To identify any additional relevant articles, citation tracking was performed by examining the reference list of the identified studies, tracking citations, and exploring related articles. Additionally, a follow-up search of gray literature sources was conducted. For more specific details and a comprehensive overview of the search process, please refer to Supplementary File.

### 2.2. Study Selection and Data Extraction

We included all studies that reported the safety, immunogenicity, effectiveness, or efficacy of mpox vaccines. Our inclusion criteria aimed to cover a wide range of articles, including original studies of various designs, such as randomized control trials (RCTs), nonrandomized control trials, and observational studies. We did not impose any restrictions on the publication date or study design. Furthermore, we considered studies involving individuals of all ages, genders, ethnicities, and geographic locations. Specifically, we focused on studies that investigated both immunizations with smallpox or mpox vaccines and the incidence of mpox. Our exclusion criteria consisted of non-human or in vitro studies, modeling studies, journal articles that were not written in English, conference papers, abstracts, author responses, books, and reviews. We also excluded articles that provided inadequate or overlapping data. All citations were imported into one Endnote library and duplicate citations were removed. Then, the citations were exported to an Excel sheet file for a two-stage screening process: (a) initial title and abstract screening; (b) full-text screening. Any conflicts were resolved by a third expert reviewer (RMG).

Four authors individually screened titles and abstracts (HA, EH, AG, and EE), who excluded non relevant articles. Editorials, reviews, letters, and abstracts only were excluded but screened for potential additional references. Full-text eligibility was conducted by (HA, EH, AG, EE, and MH) and the discrepancies were addressed in study judgments. Four reviewers independently retrieved essential data, extracted from the eligible articles. Data extraction and analysis were performed by (NY, HE, RE, NF, EE, MT) and independently verified by (RMG, MH).

### 2.3. The Outcomes and Definitions

#### 2.3.1. Primary Outcome

To assess mpox vaccines’ safety, immunogenicity, efficacy, and effectiveness.

#### 2.3.2. Secondary Outcomes

- Secondary attack rate following vaccination and reduction in disease severity in terms of hospitalization and severe symptoms.
- Assess the primary outcomes across the different routes of vaccine administration: intramuscular (IM), intradermal (ID), and subcutaneous (SC) routes.
- Assess the primary outcomes with different numbers of received doses of vaccines: one dose, two doses, and three doses.
- Address the duration of immunological response following vaccination.
- Highlight the effect of different formulations on vaccine immunogenicity.
- Evaluate the cross-reactivity of different vaccines used to prevent other OPXVs from providing protection against mpox.

In the context of the study, several key terms were defined, as follows:

**(1)**  **Vaccine safety:** The assessment of mpox vaccine safety involves the reporting and monitoring of any local or systemic adverse events that occur within 7 days after vaccination or during other specified periods, depending on the specific vaccine being evaluated.
**(2)**  **Vaccine immunogenicity:** Immunogenicity refers to the ability of the mpox vaccine to stimulate an immune response in the individual who receives it. It measures the mpox vaccine’s effectiveness in generating an immune reaction, such as the production of antibodies or the activation of specific immune cells, to protect against the target disease.
**(3)**  **Vaccine efficacy:** Vaccine efficacy is a measure of how well the mpox vaccine prevents the incidence of the disease, typically under controlled and ideal conditions. It is determined through RCTs by comparing the incidence of the disease in a vaccinated group with that in a placebo-controlled group.
**(4)**  **Vaccine effectiveness:** Vaccine effectiveness refers to the performance of the mpox vaccine in real-world conditions, beyond the controlled environment of RCTs. It assesses how well the vaccine performs in preventing the target disease within the general population through observational studies and population-based analyses.

### 2.4. Assessment of the Study Quality

The quality of non-randomized studies was assessed using the Newcastle-Ottawa Scale (NOS). According to the NOS scoring system, studies were classified as follows: Very good studies: scored 9–10 points on the NOS; good studies: scored 7–8 points on the NOS; satisfactory studies: scored 5–6 points on the NOS; unsatisfactory studies: scored 0–4 points on the NOS [31]. For non-randomized trials, we used the (ROBINS-E) tool and [32] we used the Cochrane risk of bias tool for the assessment of the quality of RCTs [33] (Supplementary File).

### 2.5. Vaccine Formulations

There are various vaccine formulations available, each with its own unique characteristics and nomenclature. One commonly used vaccine is ACAM2000, which contains live vaccinia virus and is considered a replication-competent smallpox vaccine. ACAM2000 was developed as a successor to the previously used Dryvax vaccine. Dryvax, an attenuated strain of vaccinia virus, was historically utilized for smallpox immunization. ACAM2000 differs from Dryvax in terms of its manufacturing process and production in cell culture rather than in calves. Another notable smallpox vaccine is MVA-BN (Modified vaccinia Ankara-Bavarian Nordic), which is a highly attenuated strain of vaccinia virus. MVA-BN has undergone extensive passaging to achieve further attenuation. This vaccine has been licensed under different names in various countries. In the USA and Europe, it is known as JYNNEOS and Imvamune, respectively. In some countries, it is also marketed as Imvanex. These licensed products of MVA-BN are based on the same strain but may have minor formulation differences or variations in regulatory approval for use in specific regions.

## 3. Results

**Figure 1:**
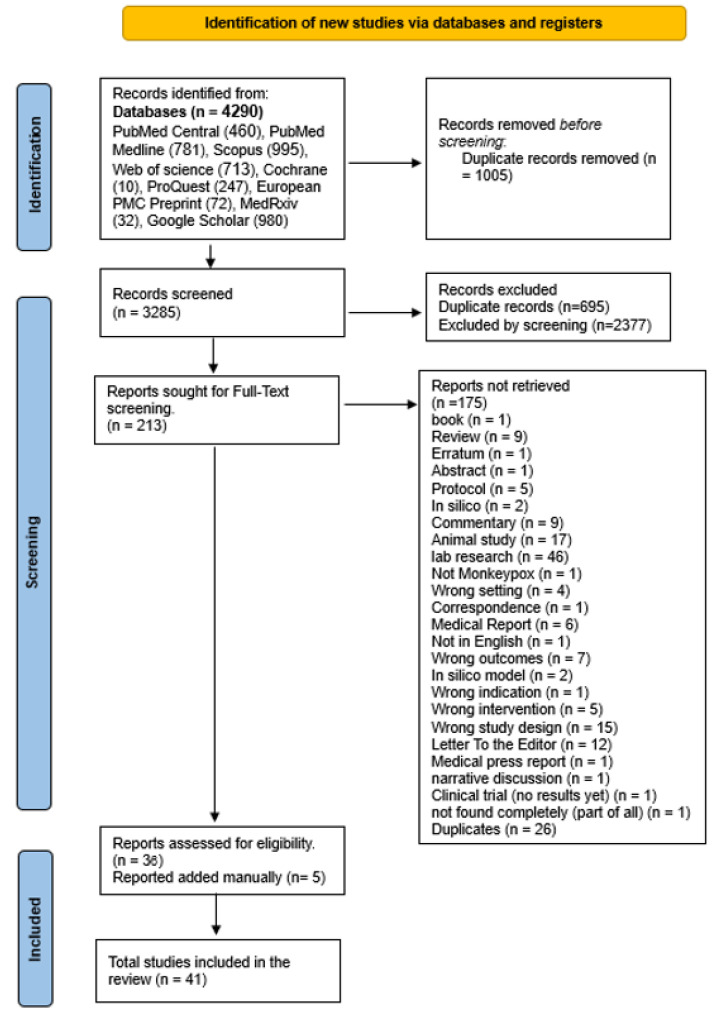
PRISMA 2020 flow diagram for updated systematic reviews which included searches of databases and registers only.

A total of 4290 citations were retrieved for screening. The number of citations obtained from the searched databases were as follows: PubMed Central (n = 460); PubMed Medline (n = 781); Scopus (n = 995); Web of Science (n = 713); Cochrane (n = 10); ProQuest (n = 247); two preprint databases—European PMC Preprint (n = 72) and MedRxiv (n = 32); and Google Scholar (n = 980). We removed 1005 citations as they were found to be duplicates by Endnote. A total of 3285 records were eligible for title and abstract screening; of these, 3072 were excluded (duplicate records (n = 695), irrelevant citations (n = 2377)). After conducting a full-text screening, out of 213 records, 177 studies were irrelevant to the research objective and were consequently excluded. From the remaining 36 studies, an additional 5 were identified through manual searches. Ultimately, 41 articles met the criteria for inclusion and were eligible for qualitative synthesis (Figure 1).

### 3.1. Summary of the Included Studies

Table 1 shows the characteristics of the included studies. One study was conducted across countries [34]; one study across Europe [35]; twenty studies in USA [26,33,36,37,38,39,40,41,42,43,44,45,46,47,48,49]; four studies in Democratic Republic of the Congo (DRC) [50,51,52,53]; two studies in Israel [54,55], Netherlands [56,57], United Kingdom (UK) [58,59], and France [60,61]; one study in Congo [62], China [63], Italy [64], Germany [65], Russia [66], Australia [67], and Spain [68]. All studies except three [57,69,70] were peer-reviewed. Most of the cases were observational studies, with the exception of a few RCTs [26,38,39,69,71]. The sample size ranged between 16 [70] and 10,915 [48]. Three studies were conducted on HCWs [53,55,64]. Finally, some studies included males only [47,54,58,68].

### 3.2. The Overall Mpox Vaccines Efficacy and Effectiveness

Almost all of the studies reported positive findings of the vaccination against mpox, with the exception of five studies. Cohn et al. [70] discovered that recombinant mpox proteins and native proteins barely bound to the JYNNEOS vaccina sera. Furthermore, vaccination with JYNNEOS does not stimulate a significant B cell response. In comparison to the vaccination, recent mpox infection (within 20–102 days) produced strong serum antibody responses to A29L, A35R, A33R, B18R, and A30L, as well as to native mpox proteins. However, those who received the JYNNEOS vaccine displayed CD4 and CD8 T cell responses against orthopox peptides that were equivalent to those seen following mpox infection. Similarly, Zaeck et al. [56] found that in non-primed people, the two-shot MVA-BN immunization series results in comparatively low levels of MPXV-neutralizing antibodies (Nabs). Karem et al. [37] concluded that systemic mpox infection is not fully protected against smallpox immunization. Catala et al. [68] revealed that there was no difference in the extension or number of lesions between patients who had received a smallpox vaccination or not in terms of the risk factors for severity. One dose of MVA-BN for PEP was ineffective in preventing symptomatic mpox in 10% of vaccinated people. The clinical illness appeared within 21 days of exposure, even with PEP, with contact with an mpox case [60].

### 3.3. Immunogenicity

Seven studies addressed the immunogenicity of different mpox vaccines. Protection was associated with the prior induction of Nabs to MVA or vaccinia virus [40]. Clinical and virologic protection against the Dryvax challenge is achieved through MVA immunization. Similarly, LC16m8 generates Nabs antibody titers to multiple poxviruses, including vaccinia, mpox, and variola major, and broad T-cell responses [42]. 92% of IMVANEX recipients and 97% of MVA-HIV recipients had anti-MVA antibodies up to 12 weeks after the illness started [61]. Based on vaccines against the vaccinia virus (VACV), it was expected that the cellular immunity induced by these vaccinations against MPXV-2022 would resemble that observed for the first-generation vaccines against MPXV-CB (Congo Basin lineage) [59]. In comparison to natural infection, patients who received the JYNNEOS vaccine showed equivalent CD4 and CD8 T cell responses to orthopox peptides. However, JYNNEOS immunization does not elicit a robust B cell response [70]. Sammartino et al. [64] found that, in naïve subjects, a second dose boosts the serological response to levels similar to those of the MPXV infected patients. Of note, the production of antibodies differed across ages: NAbs titer of 1/20 or more in 33.3 to 53.2% of people older than 45 years. Among people 30–45 years old who probably have not been vaccinated, the proportion with virus NAbs ranged between 3.2 and 6.7% [66].

### 3.4. Safety and Adverse Events

Safety and adverse effect were reported in five studies. Kennedy et al. [42]. reported that the local and systemic effects following LC16m8 immunization were comparable to those described with Dryvax. For either vaccine, there were no clinically significant abnormalities suggestive of heart damage. Greenberg et al. [43] found that MVA is a safe smallpox vaccine, even for immunocompromised individuals. The third study conducted by Duffy [44] during the 2022 mpox outbreak, found that monitoring of the JYNNEOS vaccine’s safety in the USA did not reveal any fresh or unanticipated safety issues among adults. Sharff et al. [49] reported 10 cardiac Adverse Events of Special Interest (AESI) out of 3236 doses following JYNNEOS vaccination, with an incidence of 3.1 per 1000 doses. Regarding local solicited adverse events (AEs), 91.2% of all participants experienced these adverse events after the formulation of MVA-BN by. The most common local solicited AEs were injection site pain and injection site erythema. During the entire immunization period, 69.6% of all individuals reported experiencing general solicited adverse events. Myalgia, tiredness, and headache were the most often reported general requested AEs. Nine subjects (0.8%) reported a total of nine serious adverse events (SAEs). Six individuals reported a total of eight cardiac-related adverse events of special relevance over the entire immunization period [26].

### 3.5. Vaccine Efficacy and Effectiveness

Regarding vaccine efficacy, Whitehouse et al. [62] described the incidence of confirmed mpox as being almost three-times higher among those presumed unvaccinated than among those presumed vaccinated. Arbel et al. [54] found that 3 vaccinated individuals did not have MPXV infection, but 15 unvaccinated people did (40.0 per 100,000 person-days) (6.4 per 100,000 person-days) 79% (95% CI: 24–94%). A 79% decrease in the likelihood of infection was found in those who are susceptible to MPXV infection. According to Rimoin et al. [51], the vaccine’s effectiveness was estimated to be 80.7% (95% confidence interval: 68.2–88.4%).

Few studies assessed the secondary attack rate and risk of hospitalization. The secondary attack rates among unvaccinated and vaccinated contacts were 0.017 and 0.004, respectively [50]. Immunizations resulted in decreased hospitalization rates (2%) compared to unvaccinated patients (8%). Vaccinations also reduce the incidence of systemic symptoms such as fever and chills [45].

### 3.6. Route of Administration Intradermal, Intramuscular, Subcutaneous

MVA-delivered intradermal (ID) exhibits immune and protective responses comparable to those of a 10-fold greater dosage administered subcutaneously (SC) [40]. Furthermore, ID immunization with MVA elicits similar antibodies to those elicited by the IM or SC routes but at a 10-fold-lower dose [41]. Frey et al. [38] found that there were no significant differences between IM or SC routes for IMVAMUNE, except for in induration. It was found that all routes of MVA produced binding antibodies to the whole virus, as well as NAbs to the internal mature virion and extracellular enveloped virion forms of the vaccinia virus [41].

### 3.7. Number of Doses

#### 3.7.1. Single Dose

Many studies assessed the protective effect of one dose of mpox vaccination. In the high-risk population, a single dosage of SC MVA-BN is connected to a significantly reduced probability of MPXV infection [55]. Of 10,068 individuals who received the first dose of the MVA-BN vaccination, 15 (0.15%) developed mpox subsequently. All of the identified individuals were gay and bisexual men (GBM), with 12/15 (80%) on PrEP and 3/15 (20%) being People Living With HIV (PLWH) [59]. Furthermore, a single SC MVA-BN dose is associated with a considerably reduced probability of MPXV infection in the high-risk cohort [58]. It was discovered that a dose of the JYNNEOS vaccine can decrease the severity of mpox disease in people who contract it after receiving the vaccine. Some symptoms were reported less frequently among vaccinated than unvaccinated mpox patients. Gushchin et al. [66] studied the optimal duration following the first dose of vaccination to provide protection against MPXV infection. Within two weeks of receiving the first dose of MVA-BN, the majority of postvaccination mpox infections occurred before complete efficacy was expected to have been reached.

#### 3.7.2. One Dose versus Two Doses

T cell responses were retorted after two MVA doses. The MVA immunization boosts the safety and immunogenicity of subsequent Dryvax^®^ vaccinations. It was both safe and immunogenic [39]. The immunogenic response among immunocompromised patients was addressed by Greenberg et al. [43]. The antibody responses of the HIV-positive and uninfected populations to NAbs did not differ significantly. There was only one significantly decreased total antibody titer at two weeks after the second dose of vaccination. Following immunization, MVA dramatically boosted the antibody responses in the subjects.

The single-dose effectiveness was 35.8% (95% CI, 22.1–47.1), while the two-dose effectiveness was 66.0% (95% confidence interval [CI], 47.4–78.1) [48]. The mpox incidence rate ratio (IRR) was higher among those who had not had vaccinations compared to those who had received doses 1 and 2 of the JYNNEOS vaccine over the previous 14 days (IRR = 7.4; 95% CI = 6.0–9.1 and 9.6; 6.9–13.2) [47].

#### 3.7.3. Third Dose

A third vaccination with the same MVA-based vaccine considerably increases the antibody response, but dose-sparing of an MVA-based influenza vaccine results in reduced MPXV-Nabs levels [56]. After the MVA-MERS-S vaccine, only one participant experienced cross-reactive mpox virus NAbs; following the second dose, three out of ten and the third dose, ten out of ten, respectively, experienced this reaction. On the other hand, Frey et al. [38] reported for the IMVAMUNE vaccination, a dose-response was visible for the first and second doses, but not for the third.

### 3.8. Duration of Serological Response

The duration following vaccination was important to determine the immunogenic response; two weeks following the second vaccination, the Nabs Geometric Mean Titers (GMTs) had increased significantly. For both Nabs and total antibodies, the seroconversion rates were higher than 98.0% in all groups two weeks after the second vaccination [26]. Previously naïve and vaccinated participants generated vaccinia virus and MPXV-NAbs in response to the JYNNEOS vaccination. Most participants remained IgG seropositive at the 2-year timepoint. Similarly, Priyamvada et al. [53] found that the antibody titers were strongly boosted by vaccination (peak at D42) but declined to baseline levels two years post-vaccine. Falvi et al. [69] discovered that although MVA-specific NAb IgG titers decreased over the course of five months following the second vaccination, they remained above baseline.

### 3.9. Formulation

Compare the stability, immunogenicity, and safety of the normal MVA formulation, dosage, and mode of administration with those of a more stable, lyophilized formulation and an intradermal ID route that spares antigens. Only after the initial vaccination did moderate/severe functional, local reactions substantially differ between the lyophilized-SC (30.3%), liquid-SC (13.8%), and liquid-ID (22.0%) groups. After receiving any vaccination, the Liquid-SC group (58.1%), the Lyophilized-SC group (58.2%), and the Liquid-ID group all experienced moderate-to-severe quantifiable erythema and/or induration (58.1%). In addition, 36.1% of the participants in the ID group experienced temporary, minor skin darkening at the injection site. The GMTs of the peak NAbs titers for the Lyophilized-SC, Liquid-SC, and Liquid-ID groups, respectively, were 87.8, 49.5, and 59.5 after the second vaccination day (42–208), and the maximum proportion of responders based on peak titer in each group was 97.9%, 95.3%, and 194.5%, respectively. Only 54.3%, 39.2%, and 35.2% of individuals were still seropositive for the lyophilized-SC, liquid-SC, and liquid-ID groups 180 days after the second vaccination, when the geometric mean NAbs dropped to 11.7, 10.2, and 10.4, respectively [71].

### 3.10. Cross-Reactivity

Ten studied focused on the cross-reactivity of pervious small pox vaccination on acquiring mpox. Ilchmann et al. [35] found that NAbs geometric mean titers increased after vaccination among naïve participants and among those who received a prior smallpox vaccination. Kennedy et al. [42] found that LC16m8 generates Nabs antibody titers to multiple poxviruses, including vaccinia, mpox, and variola major, as well as broad T-cell responses. Zeng et al. [63] found that only 26.7% of the sera collected from individuals born ≤1981 were positive using the MPXV IgG Elisa kit, which is consistent with MPXV Nabs.

Rimoin et al. [51] reported that the risk of human mpox is inversely associated with smallpox vaccination. Similarly, Sharff et al. [49] found that smallpox vaccination may confer cross-protection to mpox; 8.5% of the vaccinated subjects had detectable antibodies against mpox. Thornhill et al. [34] found that among 528 MPXV-infected patients, 9% had received the smallpox vaccination. The vaccine effectiveness of the prior first-generation smallpox vaccine against more severe mpox was 58% [57]. However, Huhn et al. [36] highlighted that previous smallpox vaccination was not associated with disease severity or hospitalization.

According to Fine et al. [50], immunization offered a high level of protection to the 70% of contacts who had already received vaccinations. A degree of cross-protection against mpox may be granted by historical vaccination against smallpox. Jezek et al. [52] reported that the second attack rate for contacts without a vaccination scar (7.2%) was significantly different from the attack rate for individuals who had previously had a vaccination (0.9%).

## 4. Discussion

In this review, our goal was to provide a clear understanding of the safety, immunogenicity, efficacy, and effectiveness of various mpox vaccines. In addition, we aimed to determine the second attack rate following vaccination, disease severity, and hospitalization following vaccination and assess the primary outcomes across different routes of vaccination, and the number of doses received. Finally, we highlighted the cross-reactivity of different vaccines against mpox and the duration of the immunological response, with a special focus on vaccine formulation. We analyzed a total of 41 studies to gather the relevant information. It is important to note that immune responses to one OPXV can also recognize other OPXV. The level of protection provided may vary depending on the similarity between the OPXV. With the eradication of smallpox and the subsequent discontinuation of smallpox immunization, there has been a significant rise in mpox cases. This can be attributed to several factors. Firstly, the growing population consists of individuals with limited immunological experience because of the absence of smallpox and the discontinuation of vaccination since 1980. Additionally, the increase in the number of newly infected individuals can be attributed to genetic variations within the pathogen itself and the substantial influx of travelers. Furthermore, increased human exposure to animals has occurred due to deforestation, population migration, and armed conflicts [14,73,74].

### 4.1. Vaccine Efficacy Is Variable Based on the Vaccine Type

Previous studies have demonstrated that the severe complications and sequelae associated with mpox are more common in unvaccinated than in vaccinated patients [11]. Participants who had no prior history of smallpox vaccination or exposure had lower baseline antibody levels but saw a comparable fold-rise in anti-body titers by day 42 as those who had a prior history of immunization. In response to JYNNEOS immunization, both previously unvaccinated and vaccinated subjects produce vaccinia virus and MPXV-Nabs. Finally, even while the overall mpox-specific IgG titers and Nabs titers fell from their peak and rebounded to near baseline levels by the 2-year mark, the majority of patients remained IgG seropositive [53]. Smallpox and mpox vaccinations can be administered before exposure to prevent infection and disease, or after exposure to treat infection and disease. Pre-exposure immunization is recommended to protect the most vulnerable people. This level of protection is best achieved with a second- or third-generation vaccination. Post-exposure immunization should be delivered after 4 days of exposure to prevent illness, but it can be used up to 14 days later to reduce disease severity. Post-exposure vaccination is likewise most effective with a second- or third-generation vaccine [75]. Among the first 1000 mpox cases in the Netherlands, the prior first-generation smallpox vaccine had 58% (95% CI 17–78%) effectiveness against more severe mpox symptoms, implying moderate protection against more severe mpox symptoms in addition to any possible protection against mpox infection and disease [57]. 10% of vaccinated contacts of mpox cases did not experience symptomatic mpox immunity following a single MVA-BN dose of PEPV. The symptomatic disease developed after contact with a mpox case within 21 days after exposure. This highlighted the value of the avoidance of exposure to MPXV as the vaccination does not provide 100% protection when given either pre- or post-exposure.

### 4.2. Efficacy and Effectiveness of Monkeypox Vaccines

It has been demonstrated that a single immunization dose protects against MPXV and lessens the severity of symptoms. Before full efficacy was likely, the bulk of postvaccination mpox infections happened within two weeks [55]. Some cases that arose between 1 and 14 days after vaccination may not represent true vaccine failure because the people involved sought immunization after realizing that they had been exposed because the incubation period for mpox is 7 to 21 days. After the second vaccination dosage, the protection was more noticeable. A third dose was permitted to boost protection.

During mpox vaccine trials, one potentially confusing situation arose. A few reports of outbreaks of mpox, particularly in Africa, have noted diagnostic confusion with chickenpox, another viral disease (varicella). The appearance of the exanthems might be extremely similar. On occasion, a population has been infected by two viral infections simultaneously. If a patient was exposed to mpox and received the immunization but developed a rash afterward, they would be considered vaccine failures. However, even if the rash had been misidentified as chickenpox, the mpox immunization would still have been successful [76].

### 4.3. Vaccine Safety

When compared to other replication-competent vaccinia-based smallpox vaccines, MVA-BN offers an improved safety profile as it has been attenuated to the point where it cannot replicate in mammals. Other live vaccines, including ACAM2000, have been associated with increased rates of acute myo-/pericarditis (1:200) and adverse effects relating to the heart, like dyspnea at rest (1:100) [24,77,78]. The MVA-BN vaccine thereby addresses a number of safety issues that have restricted the use of earlier versions of smallpox immunizations. No significant AEs linked to the vaccine support MVA-outstanding BN’s safety record. In stark contrast to what has been observed with replicating smallpox immunizations, Overton et al. [26] discovered that no cardiac inflammatory problems were recorded in any participant who had received the MVA-BN vaccination [24,27,77,78]. Similar to other MVA-BN investigations, local and general responses that were typically mild to moderate in intensity and self-limiting were the most often reported adverse effects following the administration of FDMVA-BN. The incidence has significantly dropped, which may be due to a number of factors, such as behavioral modifications and a gain in immunity among the susceptible population, either acquired spontaneously or as a result of targeted vaccination programs.

### 4.4. Limitations and Strengths

This study represents one of the pioneering efforts in examining the safety profile, immunogenicity, efficacy, and effectiveness of mpox vaccines. It sheds light on an important aspect of research that has been relatively scarce in the literature. A key strength of this review is the comprehensive inclusion of a large number of literature sources. The review not only encompassed published studies available in databases, but also unpublished data, such as preprints. The review included a substantial number of studies, with a total of 41 studies being selected for analysis. These studies collectively encompassed a significant participant pool. Furthermore, to ensure the integrity of the study and to minimize bias, we adhered to specific inclusion and exclusion criteria during the study selection process. However, more information about the brand name of the smallpox vaccine that was used could not be added as it was not available in the included studies. Finally, we assessed the quality of the included studies based on the study type, as recommended by the PRISMA guidelines.

## 5. Conclusions

Mpox is a growing health problem; however, there is a scarcity of studies that assess the vaccines’ safety profile, immunogenicity, safety, and efficacy. Mpox vaccines can be used to effectively prevent the disease and control its further spread. Cross-reactivity induced by smallpox vaccines provides sufficient immunity against mpox. In many studies, mpox resulted in the development of an immunological response similar to that developed by natural infection. However, there is an urgent need to conduct more research focusing on racial response differences and responses among high-risk populations to ensure the health emergency preparedness to combat this disease.

## Figures and Tables

**Table 1:** Shows the characteristics of included studies.

Author, Year, Country	Aim Study Design Study Setting Duration of the Study	Sample Size Population Criteria Inclusion Criteria Exclusion Criteria	Type of Intervention Vaccine Doses (Number)	Key Findings (Safety Efficacy Immunogenicity Effectiveness)
Fin, et al. 1988 [50] DRC	Studied the epidemiology of mpox in unvaccinated humans. Observational study Health institutions in the endemic regions. 1980–1984.	N = 209 Participants were categorized into: primary cases, co-primary cases, infectious cases, contact cases, household contacts, and secondary cases.	smallpox vaccine (vaccinia)	For contacts in houses and contacts outside of households, the rates of a second attack were 0.110 and 0.038, respectively. For contacts who had received vaccinations, the equivalent rates were 0.017 and 0.004, respectively. There are no appreciable variations in assault rates between younger and older unvaccinated children. Effectiveness was 85% for household contacts and 89% for further domiciliary contracts, for a total efficacy of 85%. Thus, the vaccine was offering a significant level of protection to the 70% of contacts who had a prior history of immunization.
Whitehouse et al. 2021 Congo [62]	Enhance mpox surveillance. Observational study Congo. January 2011–December 2015	N = 3639 Patients with confirmed mpox, irrespective of VZV status, with rash onset from January 2011 to December 2015. Median age: 14 years Males and females Different occupations	Smallpox vaccination	The incidence of confirmed case patients was nearly three-times greater in the presumed unvaccinated group than in the presumed vaccinated group, suggesting historical smallpox immunization may have some degree of cross-protection against mpox.
Rimoin 2010 [51] DRC	Risk of human mpox infection after cessation of official smallpox vaccination campaign. Cohort Kasai Oriental province November 2005–November 2006	N = 760 5–70 years Males: 62.1% Farmers and hunters PCR-based molecular assays were used for the diagnosis of mpox. Exclusion: 1. Cases without fever or rash 2. Cases with negative PCR	Smallpox vaccine	Inversely correlated with smallpox immunization is the risk of human mpox. The predicted vaccine effectiveness was 80.7% (95% CI: 68.2–88.4%).
Huhn et al. 2005 [36] USA	Knowledge of the clinical manifestations of mpox. Case-control Health care settings	N = 34 6–47 years Males and females Patients with confirmed mpox	Comparison between pediatric and adult patients and between patients with and without previous smallpox vaccination	Patients under the age of 18 were more likely to be admitted to an intensive care unit. An independent association was found between nausea, vomiting, and mouth sores and a hospital stay lasting more than 48 h and 3 abnormal laboratory tests. No patients passed away, although 5 (15%) were classified as extremely unwell, and 9 (26%) spent more than 48 h in the hospital. Hospitalization or disease severity were not related to previous smallpox immunization.
Karem et al. 2007 [37] USA	Evaluate correlations between immunological markers, smallpox vaccination status, and mpox infection outcomes. Case-control Six affected states One year following the U.S. mpox outbreak of 2003	N = 72 <31 years and >31 years of age Males and females Cases defined by standard definition and household contacts of cases. Participants were categorized into vaccinated cases, unvaccinated cases, vaccinated contacts, and unvaccinated contacts. Exclusion: 1. unknown vaccination history 2. refused to give biological tissues	Smallpox vaccine	The smallpox vaccine does not offer full defense against systemic mpox infection. In both vaccinated and unvaccinated mpox cases, anti-OPXV IgM and CD8 responses predominated, with IgG, CD4, and memory B-cell responses indicating vaccine-derived immunity. Immune indicators showed that some vaccinated people and unvaccinated people had asymptomatic illnesses. The smallpox vaccine does not offer full defense against systemic mpox infection. In both vaccinated and unvaccinated mpox cases, anti-OPXV IgM and CD8 responses predominated, with IgG, CD4, and memory B-cell responses indicating vaccine-derived immunity. Immune indicators showed that some vaccinated people and unvaccinated people had asymptomatic illnesses.
Catala et al. 2022 [68] Spain	To document the clinical and epidemiological characteristics of cases of mpox in the current outbreak. Cross-sectional Multiple medical facilities From 28 May–14 July 2022	N = 185 Median age 38.7 years Males PCR-positive mpox virus-infected patients with skin/dermatological lesions		Regarding risk factors for severity, the study found no distinction in the extension or quantity of lesions between patients who had received a smallpox vaccination or not.
Thornhill et al. 2022 [34], multi-national study	Describe the presentation, clinical course, and outcomes of PCR–confirmed monkeypox virus infections. Observational 16 countries From April–June 2022	N = 528 Males Confirmed infections diagnosed.	Smallpox vaccination	Overall, 9% said they had already been immunized against smallpox.
Jezek et al. 1986 DRC [52]	Attack rate among contact. Observational Health care report From 1980 to 1984	N = 2510 0 to 4 years 5–14 years ≥15 years Contacts to mpox-infected patients		Vaccinated individuals appeared to have an effective immunity. The attack rate among contacts without a scar from a previous vaccination (7.2%) was substantially higher than the assault rate among those who had previously received a vaccination (0.9%).
Wolff et al. 2023 [55] Israel	Effectiveness of a single, SCT dose of MVA-BN. Electronic health records Retrospective cohort Clalit Health Services (CHS) From 23 July 2022–22 December 2022	N = 2054 Male HCWs Eligible for the vaccine on 31 July 2022. Dispensed HIV-PrEP at least for 1 month, or diagnosed with HIV, and also were diagnosed with one or more STIs since 1 January 2022. Exclusion: Individuals who were infected with mpox before the study period	Single dose	A single dose of subcutaneous MVA-BN in this high-risk cohort is associated with a significantly lower risk of MPXV infection.
Hubert et al. 2023 [61] France	Levels of NAbs and MVA-Abs in previously smallpox-vaccinated individuals, mpox-infected patients, and IMVANEX or MVA-HIV vaccine recipients. Cohort Hospital Henri Mondor From 2014–2015	Uninfected donors (n = 88) MPXV-infected (n = 48) IMVANEX recipients (n = 86) MVA-HIV recipients (n = 66) Uninfected donors: 51year s MPXV-infected: 32 years IMVANEX recipients: 54 years All males except—uninfected donors Female 53 (60%)	IMVANEX vaccine two doses MVA-HIV vaccine two doses	Up to 12 weeks following the commencement of the illness, anti-MVA NAbs were found in 94% of MPXV-infected patients, 92% of IMVANEX recipients, and 97% of MVA-HIV recipients. The smallpox-vaccinated and post-mpox individuals had the greatest anti-MVA and anti-MPXV NAb titers.
Frey 2007 [38] USA	Safety and immunogenicity of IMVAMUNE. Partially blinded RCT Saint Louis University. From 17 May 2004–21 June 2005	N = 90 8–32 years Male: 58 (64.4%) vaccinia-naïve Negative: HBV HCV, and HIV Exclusion: 1. Military service prior to 1989 or after January 2003 2. CVS diseased	IMVAMUNE vaccine (2 doses) Dryvax(one dese)	- No significant differences between IM or SC routes for IMVAMUNE except for induration. - IMVAMUNE^®^ was safe and well-tolerated compared to Dryvax - IMVAMUNE limits local reactogenicity to Dryvax^®^ - A dose-response was apparent for the first and 2nd vaccination but not the third vaccination
Parrino 2007 [39] USA	Safety and immunogenicity of MVA. Randomized, placebo-controlled, double-blinded RCT National Institutes of Health (NIH) December 2002 and May 2004	VRC 201(vaccinia-naïve): 76 VRC 203(vaccinia-immune): 75 VRC 201 18–33 years (vaccinia-naïve) VRC 203 born no later than 1979 (vaccinia-immune) VRC 201: 18–33 years, without prior vaccination with any vaccinia product VRC 203: healthy volunteers, born no later than 1979 Exclusion: contraindication to receive Dryvax, history of heart	Modified Vaccinia Ankara (MVA) vaccine Vaccinia-naïve: randomized to receive one dose of Dryvax^®^ or a 0.5 mL IM injection of TBC-MVA or placebo Vaccinia-immune: randomized to receive 1 or 2 doses of TBC-MVA or placebo. 3–12 weeks later: all participants were scheduled to receive Dryvax^®^	- No serious systemic reactions were reported. - Vaccinia-naïve volunteers developed non-specific macular rash 8 days after Dryvax - T cell responses were seen after two MVA doses. MVA vaccine boosts the safety and immunogenicity of later Dryvax^®^ vaccination and is both safe and effective.
SSivapalasingan 2007 [72] USA	Cellular and humoral immunity to vaccinia virus (VV) in individuals exposed to 3 different OPXV. Cohort University of Massachusetts Medical School April 2002 and October 2005	VV vaccination (n = 154) History of Mpox (n = 7) History of variola virus (n = 8) Poxvirus-naïve (n = 15) 18–33 years Exclusion: 1. history of variola virus infection 2. history of mpox virus infection	smallpox vaccine (vaccinia)	Six of the seven individuals exhibited IFN-ELISPOT responses, all had VV-specific LP responses, and three of the seven developed VV-specific neutralizing antibodies one year after contracting the MPXV virus.
Seaman 2010 [40] USA	To examine the effect of immunization with MVA upon challenge with replication-competent vaccinia (Dryvax). Clinical Trial From 2006–2007	N = 36 20–34 years Males 18 (50%) Healthy men or women, at least 18 years of age, and had no history of smallpox vaccination. Exclusion: Diseased individuals or a history of smallpox vaccination	Two doses of MVA	Clinical and virologic protection against the Dryvax challenge is achieved by MVA immunization. Prior introduction of NAbs to MVA or vaccinia virus is linked to protection. ID delivered MVA induces immunologic and protective responses comparable to those induced by SC administration of a 10-fold greater dosage. By day 7, MVA patients developed NAbs titers that could be detected. The 1 × 108 subcutaneous, 1 × 107 intradermal, 1 × 107 intramuscular, and 1 × 107 subcutaneous groups all saw peaks in their responses on days 14 and 28. On day 14, the two participants in the 1 × 106 ID group’s anti-vaccinia virus NAbs responses were less intense than those in the other MVA vaccine groups, but by day 28, they were comparable.
Wilck 2010 [41] USA	Safety and immunogenicity of MVA (ACAM3000 MVA). Clinical Trial From October 2005–March 2007	N = 72 18–34 years Females: 43 (59.7%) Healthy men or women who were at least 18 years of age, were born after 1971 and had no history of smallpox vaccination. Exclusion: Diseased individuals or history of smallpox vaccination.	Two doses of MVA	Similar antibodies to those produced by the IM or SC methods are produced after ID immunization with MVA, but at a dose that is ten times lower. All dose levels and administration methods for MVA were well tolerated. The ID and SC routes showed more pronounced local reactogenicity than the IM route. All means of administering MVA resulted in the induction of binding antibodies to the whole virus as well as NAbs to the internal mature virion and extracellular enveloped virion forms of the vaccinia virus. These responses were stronger for the higher dose administered via each route. An interferon g enzyme-linked immunospot assay was used to determine the T-cell immunological responses to the vaccine virus, however there was no obvious correlation between the dose or delivery method.
Kennedy, 2011 [42] USA	Compare the safety and immunogenicity of LC16m8 with Dryvax in vaccinia-naive participants. Phase I/II clinical trial Five sites inside the USA From October 2004–June 2005	N = 154 18–34 years Males: 97 (63%) Healthy vaccinia-naive adult volunteers (year of birth, 1971–1987) with negative serology for hepatitis B and C and human immunodeficiency virus, negative urine glucose, and a normal ECG Exclusion: History of smallpox vaccination or diseased individuals	LC16m8 with Dryvax Single dose	Broad T-cell responses and Nabs antibody titers against many poxviruses, including vaccinia, mpox, and variola major, are produced by LC16m8. Local and systemic effects of the LC16m8 vaccine were similar to those that were reported after receiving Dryvax. For either vaccine, there were no clinically significant abnormalities that were indicative of cardiac damage. Antivaccinia, antivariola, and antimpox Nabs titers were obtained with both vaccines. 1:40, despite the fact that Dryvax considerably outperformed LC16m8 in terms of mean plaque reduction, neutralization titers at day 30 following vaccination for anti-NYCBH vaccinia, anti-mpox, and antivariola. Strong cellular immune responses from LC16m8 trended greater than those from Dryvax for lymphoproliferation but lower than those from IFN-c ELISPOT.
Greenberg 2013 [43] USA	Evaluate the safety of MVA and immunogenicity in HIV-infected and uninfected subjects. phase I/II clinical trial 5 USA centers	N = 151 Male18–49 years Nonpregnant women 18–55 years vaccinia experienced or vaccinia naive previous smallpox vaccination. HIV-infected participants had to be receiving stable or no HAART for >6 months prior to enrollment. Exclusion: 1. ≥10% risk of developing myocardial infarction or coronary death within 10 years. 2. an immediate family member with the onset of IHD before age 50 years 3. a history of active AIDS, diabetes, malignancy, organ transplantation, or clinically significant and severe illness.	Two doses of MVA Vaccination	Even for those with impaired immune systems, MVA is a safe smallpox vaccine. There was just one significantly reduced total antibody titer at 2 weeks following the second vaccination, and there were no significant differences between the antibody responses of the uninfected and HIV-infected populations for NAbs. In subjects who had already been exposed to vaccinia, MVA significantly increased the antibody responses, demonstrating its potency against variola.
Frey 2015 [71] USA	Compare the safety and immunogenicity of the standard formulation of MVA, dose, and route with both a more stable, lyophilized formulation and with an antigen-sparing intradermal ID route of administration. Phase II RCT 8 sites inside the USA From 9 February–2 September 2010.	N = 524 18–38 years Males 49.6% Healthy, born after 1971, not pregnant, and had an acceptable ECG, ≤10% risk of myocardial infarction or coronary death, and no evidence of a vaccinia scar, history of smallpox vaccination, or history of eczema. Exclusion: Unhealthy subjects	MVA Lyophilized-SC/Liquid-SC/Liquid-ID Two doses	Only after the initial vaccination did moderate/severe functional local reactions substantially differ between the Lyophilized-SC (30.3%), Liquid-SC (13.8%), and Liquid-ID (22.0%) groups. After receiving any vaccination, the Liquid-SC group (58.1%), the Lyophilized-SC group (58.2%), and the Liquid-ID group all experienced moderate-to-severe quantifiable erythema and/or induration (58.1%). 36.1% of the participants in the ID Group experienced temporary, minor skin darkening at the injection site. The geometric mean of peak NAbs titers for the Lyophilized-SC, Liquid-SC, and Liquid-ID groups, respectively, was 87.8, 49.5, and 59.5 after the second vaccination day (42–208), and the maximum proportion of responders based on peak titer in each group was 97.9%, 95.3%, and 194.5%, respectively. Only 54.3%, 39.2%, and 35.2% of individuals were still seropositive for the Lyophilized-SC, Liquid-SC, and Liquid-ID groups 180 days after the second vaccination, when geometric mean NAbs dropped to 11.7, 10.2, and 10.4, respectively.
SFAhmed 2022 [67] Australia	Investigate the expected cross-reactive immunity of VACV against the MPXV-2022 outbreak viruses. Cohort study Databases Genes analysis (humans) 2022		Vaccinia virus (VACV). Three major types: First-generation vaccines comprise live VACV, e.g., Dryvax Second-generation vaccines comprise live VACV, e.g., ACAM2000, 3) Third-generation vaccine, Bavarian Nordic’s MVA-BN	The genetic similarity between the VACV reference sequence and the VACV-based vaccination sequences is approximately 98%. (Dryvax, ACAM2000, and MVA-BN). These VACV-based vaccinations against MPXV-2022 were expected to generate cellular immunity similar to that shown with first-generation vaccines against MPXV-CB. There was a substantial genetic similarity between VACV and the MPXV-2022 consensus sequence and the MPXV-CB reference sequence for all eight immunogenic proteins (range: 94–98%).
R Arbel 2022 [54] Israel	Evaluate effectiveness after providing one dose of MVA to individuals at risk of MPVX infection. Cohort study Electronic medical records Clalit Health Services From 31 July 2022–12 September 2022	N = 1970 18–42 years Males Dispensed HIVPrEP at least for one month, or who were diagnosed with HIV and also were diagnosed with one or more of the following STIs since 1 January 2022 (Active Syphilis, Chlamydia, or Gonorrhea) Exclusion: Participants were vaccinated after 18 August 2022, and those who were infected with MPXV before the study period.	One dose of Modified Vaccinia Ankara (MVA)	Overall, 44% received the MVA vaccine and followed up for at least 25 days. Infections with MPX occurred in 15 subjects who were unvaccinated (40.0 per 100,000 person-days) and in 3 participants who were vaccinated (6.4 per 100,000 person-days) (95% CI: 24–94%). a 79% decrease in the probability of infection in those who are susceptible to MPX infection.
Bertran 2022 [58] UK	Assess the effectiveness of MVA–BN against laboratory-confirmed symptomatic mpox disease in the GBMSM. Observational study Primarily sexual health services 21 December 2022	N = 363 5 years and older Males Only cases with an index date from 4 July–9 October 2022 Exclusion: Females, self-reported heterosexual men, and those with missing vaccination information.	A single dose of Modified Vaccinia Ankara–Bavaria Nordic (MVA–BN)	A single MVA–BN dose was highly protective against symptomatic mpox disease among at-risk GBMSM.
Duffy 2022 [44] USA	To detect adverse events after vaccination Cross-sectional survey Datalink health surveillance systems From 22 May–21 October 2022	N = 1350 All ages Males and females Vaccine recipients of all ages	JYNNEOS 0.1 mL doses by ID injection for adults aged ≥18 years SC 0.5 mL doses for persons aged <18 years	During the 2022 mpox outbreak, monitoring of the JYNNEOS vaccine’s safety in the US has not revealed any fresh or unanticipated safety issues among adults.
Farrar 2022 [45] USA	Describe the Demographic and Clinical Characteristics of Mpox of cases occurring ≥14 days after receipt of 1 dose of JYNNEOS vaccine and compared to unvaccinated persons. Cross-sectional survey 29 U.S. Jurisdictions From 22 May–3 September 2022	N = 6606 ≥18 years Males and females 276 mpox cases who received 1 dose of JYNNEOS vaccine ≥14 days before illness onset and 6329 unvaccinated. Exclusion: No available vaccination information	A single dose of the JYNNEOS Vaccine	People who contract mpox after receiving a dose of the JYNNEOS vaccine may experience less severe symptoms. Compared to mpox individuals who were unvaccinated, several symptoms were observed less frequently. Hospitalization rates for vaccinated individuals were lower than those for unvaccinated patients (8%) at 2%, and systemic symptoms like fever and chills were less common among vaccinated patients.
VAGushchin 2022 [66] Russia	To study residual immunity, Serum samples were examined for the presence of IgG antibodies against the Vaccinia virus, as well as for the ability to neutralize plaque formation with the Vaccinia virus MNIIVP-10 strain. Cross-sectional study Moscow From 2–16 June 2022	N = 2908 30–80 years Males and females People living in the city of Moscow. Exclusion: No age information	Vaccinia administration	A titer of 1/20 or higher is detected in 33.3–53.2% of those over the age of 45. The percentage of people having viral NAbs among 30- to 45-year-olds who are likely unvaccinated ranged from 3.2–6.7%. Despite having higher levels of antibodies, those beyond the age of 66 had a somewhat lower percentage of positive samples than those between the ages of 46 and 65.A titer of 1/20 or higher is detected in 33.3–53.2% of those over the age of 45. The percentage of people having viral NAbs among 30- to 45-year-olds who are likely unvaccinated ranged from 3.2–6.7%. Despite having higher levels of antibodies, those beyond the age of 66 had a somewhat lower percentage of positive samples than those between the ages of 46 and 65.
Hazra 2022 [66] USA	To describe mpox infections after a single dose of MVA-BN. Cohort study Electronic medical record From 28 June–9 September 2022	N = 90 All ages Males and females Patients who tested positive for mpox at least 1 day after receiving the first dose of the MVA-BN Exclusion: Not vaccinated or no vaccination status	A single dose of MVA-BN, JYNNEOS	The bulk of post-vaccination mpox infections happened within two weeks of receiving the first dose of MVA-BN before complete efficacy was probably attained.
Merad 2022 Lyon, [60] France	Determine the outcomes of at-risk contacts of mpox cases vaccinated with a single MVA-BN dose given post-exposure. Cohort study Auvergne-Rhône-Alpes region. From 15 June–12 August 2022	N = 108 29–44 years 97 men (90%), 11 women (10%) Vaccinated with one dose of MVA-BN (IMVANEX) as part of post-PEPV. Exclusion: Mpox symptoms documented before vaccination. Time from contact to vaccination >14 days Missing data, withdrew consent	One dose of MVA-BN ≤ 14 days post-exposure	Overall, 10% of vaccinated contacts of mpox cases did not have symptomatic mpox after receiving a single MVA-BN dose of PEPV. Notwithstanding PEPV, a symptomatic illness appears in contact with a case of mpox within 21 days of exposure.
Payne 2022, USA [46]	Examine the JYNNEOS vaccination’s protection against mpox. Cross-sectional survey 43 USA jurisdictions From 31 July–1 October 2022	N = 9544 18–49 years Male Unvaccinated or had received either 1 or 2 JYNNEOS doses, and reported mpox	1st and 2nd doses of JYNNEOS vaccine	Mpox incidence estimates were higher among those who had not received vaccinations compared to those who had received only one dose of the JYNNEOS vaccine within the previous 14 days (IRR = 7.4; 95% CI = 6.0–9.1) and those who had received dose two within the previous 14 days (IRR = 9.6; 95% CI = 6.9–13.2).
Payne 2022 [47] USA	Examine the incidence of mpox among unvaccinated persons and those who had received ≥1 JYNNEOS vaccine dose as PEP. Cross-sectional study 32 USA jurisdictions From 31 July–3 September 2022	N = 5402 18–49 years Males Reported mpox	1st and 2nd doses of JYNNEOS vaccine	The average mpox incidence (cases per 100,000) was 14.3 (95% CI = 5.0–41.0) times higher in unprotected individuals than it was in those who had received one dose of the JYNNEOS vaccine 14 days prior.
LPriyamvada 2022 [53] DRC	Assess the quality and longevity of serological responses to two doses of JYNNEOS vaccine. Cohort study Kinshasa and Tshuapa Province in the DRC	N = 999 18 years or older Both sex HCWs Exclusion: Pregnant women	Two doses of JYNNEOS then, the IMVAMUNE vaccine	Participants who have received vaccinations before and those who have not produced MPXV-NAbs and vaccinia virus in response to JYNNEOS immunization. At the 2-year timepoint, the majority of subjects are still IgG seropositive. The substantial increase in antibody titers was caused by immunization (Peak at D42), but two years after the vaccination, fall to baseline levels
VanEwijk 2022 [57] Netherlands	Public health response, epidemiological and clinical characteristics of the first 1000 cases and protection of the first-generation smallpox vaccine. Observational study From 20 May–8 August 2022.	N = 1000 31–45 years Males and females Cases were reported as confirmed, probable, and possible cases. Confirmed MPX cases were categorized into mild or more severe MPX		Vaccine effectiveness of the prior first-generation smallpox vaccine against more severe MPX of 58% (95% CI 17–78%).
Agunbiade 2023 [59] UK	Clinical characteristics of mpox infection in individuals after 1st dose of MVA Cohort study Health clinics part of Chelsea and Westminster Hospital NHS Foundation Trust, in London (UK) From 20 June–31 October 2022	N = 10,068 Gay, bisexual, and other men who have sex with men (GBM) Confirmed infection by RT-PCR and received a single dose of (MVA-BN) at least 1day prior to the onset of mpox-associated symptoms	Single dose MV	Of the 10,068 individuals who received the first dose of the MVA-BN vaccination, 15 (0.15%) developed mpox subsequently. All individuals identified were GBM with 12/15 (80%) on PrEP and 3/15 (20%) PLWH.
Cohn 2023 USA [70]	Examine the polyclonal serum and single B cell antibody repertoires and T cells induced by the JYNNEOS vaccine as well as mpox infection. Cross-sectional	N = 16 (10 vaccinated with JYNNEOS, 6 infected) Vaccinated: males Infected: males (83.3%) and females Vaccinated: 21–30 years (10%), 31–40 years (40%), 41–50 years (50%) Infected: 31–40 years (50%), 41–50 years (33.3%), 51–60 years (33.3%)	1 dose and 2 doses of JYNNEOS vaccine	Gene-level plasmablast and antibody responses were negligible and JYNNEOS vaccinee sera displayed minimal binding to recombinant mpox proteins and native proteins JYNNEOS vaccine recipients presented comparable CD4 and CD8 T cell responses against orthopox peptides to those observed after mpox infection JYNNEOS immunization does not elicit a robust B cell response. Recent mpox infection: (within 20–102 days) induced robust serum antibody responses to A29L, A35R, A33R, B18R, and A30L, and to native mpox proteins compared to the vaccine.
Deputy 2023 [48] USA	Assess the effectiveness of JYNNEOS vaccination in preventing medically attended mpox disease among adults. Case-control Epic Cosmos platform, electronic health record (EHR) database From 15 August–19 November 2022	N = 10,915 (2266 case, 8649 control) All ages Males, females, and other Cases: with initial mpox diagnosis or a positive mpox laboratory test. Control patients: with HIV diagnosis or a positive HIV test, or a new or refill order for HIV PrEP Exclusion: Patients with a previous mpox diagnosis or positive MPXV laboratory test	1 dose (partial vaccination) or 2 doses (total vaccination) of the JYNNEOS vaccine	Single-dose effectiveness was 35.8% (95% CI, 22.1 to 47.1), while two-dose effectiveness was 66.0% (95% confidence interval [CI], 47.4 to 78.1)
Ilchmann 2023 [35] Europe	To assess safety, immunogenicity, and boost response with MVA-BN in healthy adults with and without prior smallpox vaccination. The initial study: phase 2, partially randomized, double-blind, placebo-controlled, noninferiority trial. The follow-up study: 2 open-label trial A single European site 3 years (2006–2009)	The initial study N = 745 Participants naive to smallpox vaccination were randomized to: 1 dose MVA-BN (n = 181) 2 doses MVA-BN (n = 183) placebo (n = 181). Participants with previous smallpox vaccination received 1 MVA-BN booster (HSPX, n = 200). The follow-up study N = 152 1 dose MVA-BD (n = 77) 2 doses MVA-BD (n = 75) 18–55 years Male and females non-pregnant women No comorbidity	Participants without prior smallpox vaccination were randomized 1:1:1 to receive vaccinations 4 weeks apart with 1 dose of MVA-BN followed by 1 dose of placebo (1 × MVA), 2 doses of MVA-BN (2 × MVA), or 2 doses of Tris buffer placebo (PBO). Participants with prior smallpox vaccination were given a single booster dose of MVA-BN. The follow-up study: participants without prior smallpox vaccination (1 × MVA BD and 2 × MVA BD groups) Participants with previous smallpox vaccination received 1 MVA-BN booster	NAbs geometric mean titers increased after vaccination among naïve and among those who received a prior smallpox vaccination.
Raadsen 2023 [65] Germany	Report that cross-reactive mpox virus NAbs. A single-center, open-label phase 1 clinical trial 2017 and 2018	N = 10 18–40 years Participants without previous VACV	MVA Encoding Middle East Respiratory Syndrome–Coronavirus Spike Protein 1st, 2nd and 3rd dose	Cross-reactive mpox virus NAbs were detectable in only one participant after the first dose of the MVA-MERS-S vaccine, in 3 of 10 after the 2nd dose, and in 10 of 10 after the third dose.
Sammartino 2023 [64] Italy	Evaluation of humoral response elicited by natural infection and healthy vaccinated subjects, including historically smallpox-vaccinated individuals and newly vaccinated subjects. Cohort study May and August 2022	N = 123 20–71 years Males and females Individuals diagnosed with MPXV infection including HCWs	Vaccinated with VACV 1st and 2nd dose.	A robust immune response brought on by the natural infection can stop the condition. A second dose increases the serological response in naive subjects to levels that are comparable to those of MPXV patients. The t-cellular response is the most obvious sign of protection in smallpox-vaccinated controls even years after vaccination.
KASharff 2023 [49] USA	Evaluate cardiac AESI following JYNNEOS vaccination; describe the incidence of cardiac AESIs in the Kaiser Permanente Northwest (KPNW) population who received a JYNNEOS vaccination. Cohort study Electronic health records From 14 July–10 October 2022	N = 2126 12 years and older Males and females The patients were vaccinated with at least 1 dose of the JYNNEOS vaccine, had been a KPNW member at the time of vaccination, and through the 21-day follow-up period	JYNNEOS vaccination	Overall, 10 cardiac AESIs were found, with a frequency of 3.1 per 1000 doses out of 3236 doses. Immunization against smallpox may provide cross-protection to the mpox 8.5% had detectable antibodies. The group of people 71 to 80 years old had the highest coverage (78%) rate. A priority should be given to their vaccination because 31.5% of this demographic have low antibody levels.
Overton 2023 [26] USA	Show the consistency of Nabs immune responses to 3 consecutively produced lots of FD MVA-BN. To assess uncommon adverse reactions, with a focus on cardiac signs and symptoms indicating myo-/pericarditis. RCT double-blind, multicenter, phase 3 trial 12 sites in the United States 2020	N = 1129 Group 1 (n = 377) Group 2 (n = 375) Group 3 (n = 37) 18–45 years Males and females Healthy non-pregnant and non-lactating women	Vaccine-freeze-dried formulation of MVA-BN 2 vaccinations doses 4 weeks apart.	Overall, 91.2% of participants reported encountering locally solicited AEs. For 87.2% and 73.2% of all individuals, respectively, injection site discomfort and injection site erythema were the most frequently reported local requested adverse events (AEs). During the entire immunization period, 69.6% of all individuals reported experiencing general solicited adverse events. Myalgia, weariness, and headache were the most typical general requested AEs, affecting 40.6–45.5% of all patients. Nine subjects (0.8%) throughout the three lots reported a total of nine serious adverse events (SAEs). Six subjects reported a total of eight cardiac-related adverse events of special interest (AESIs) throughout the entire immunization period (0.5% across the three lots). Nabs antibody GMTs had risen from undetectable two weeks after the second vaccination (at Week 6), to 252.7 for Lot Group 1, 269.9 for Lot Group 2, and 242.0 for Lot Group 3. There were no statistically significant differences between the three lot groups for neutralizing and total antibodies; however, seroconversion rates 2 weeks after the second vaccination were above 98.0% for both Nabs and total antibodies in all groups.
Zaeck 2023 [56] Netherlands	Measured MVA-reactive, VACV-reactive, and MPXV-reactive binding and neutralizing antibodies in cohorts of historic smallpox-vaccinated, MPXV PCR-positive, MVA-BN-vaccinated and MVA-H5-vaccinated individuals. Cohort study 2022	N = 238 participants sera collected in 2022 (n = 126) (n = 59 born before or during 1974 (n = 59) born after 1974 (n = 67) Healthy individuals and patients suspected of having MPXV infection		After both an MPXV infection and a previous smallpox vaccine, MPXV-Nabs can be found. In non-primed people, a two-shot MVA-BN immunization series results in comparatively modest levels of MPXV-neutralizing antibodies. Lower MPXV-Nabs levels result from dose-sparing an MVA-based influenza vaccine, but a third dose of the same MVA-based vaccine considerably increases the antibody response.
Zeng 2023 [63] China	Evaluated the Nabs level in serum samples from participants based on year of birth. Lab study using vesicle swabs of a mpox-infectious case. Hong Kong	N = 30 Individuals who were immunized (n = 15) born ≤1981 Non-immunized with smallpox vaccine (n = 15) born >1981		Only 26.7% of sera obtained from people born between 1981 and today tested positive using the MPXV IgG Elisa kit, which is consistent with MPXV Nabs. Sera that were unable to Nabs MPXV tested negative in the ELISA for MPXV IgG antibodies.
Falvi, 2022 [69] USA	Demonstrate that vaccinations of COH04S1 can elicit robust OPXV immunity. Open-label and randomized, placebo-controlled, phase 1 City of Hope (COH) as part of a clinical protocol 6 months	20, Male 8 (40%), 36y (22, 54) Inclusion: Healthy adult Exclusion: Age < 18 or >55, previous SARS-CoV-2 infection, and poxvirus vaccination	two doses of COH04S1 at days 0 and 28 low-dose (DL1, 1 × 107 pfu), medium-dose (DL2, 1 × 108 pfu), or high-dose (DL3, 2.5 × 108 pfu) of vaccine	Regardless of the dose, COH04S1 demonstrated post-vaccination increases in MVA-specific IgG, NAb titers, and significant OPXV-specific cellular responses that persisted for more than six months. Over the course of five months following the second vaccination, MVA-specific NAb and IgG titers decreased, although they remained above baseline. A seroconversion rate of 30–60% was observed in DL1 cohorts. DL2 and DL3 patients displayed 100% seroconversion following the initial dosage.

DRC: Democratic Republic of The Congo; USA: United States of America; Nabs: Neutralizing Antibodies; MVA-Abs: Modified Vaccinia Ankara-Based Vaccine; MVA-HIV: Modified Vaccinia Ankara- Human Immunodeficiency Virus; RCT: Randomized Control Trial; HBV: Hepatitis B Virus; HCV: Hepatitis C Virus; HIV: Human Immunodeficiency Virus; CVS: Cardio-Vascular System; VRC: Vaccine Research Center; HIV-PrEP: Human Immunodeficiency Virus Pre-Exposure Prophylaxis; MVA; Modified Vaccinia Ankara; VV: Vaccinia Virus; ELISPOT: Enzyme-Linked Immunospot; LP: Lymphoproliferative; IFN: Interferon; ID:Intra-Dermal; SC; Subcutaneous; HAART: Highly Active Antiretroviral Therapy; ECG: Electrocardiography; IHD: Ischemic Heart Disease; AID: Autoimmune Disease; GBMSM: Gay, Bisexual, And Other Men Who Have Sex with Men; MVA–BN: Modified Vaccinia Ankara–Bavaria Nordic; PEP: Postexposure Prophylaxis; PLWH: People Living With HIV; VACV: Vaccinia Virus; AESI: Adverse Events of Special Interest; KPNW: Kaiser Permanente Northwest; FD MVA-BN: Freeze-Dried Formulation Of MVA-BN; OPXV: orthopoxvirus.

## Data Availability

Data are available upon request by mailing the first author.

## Supplementary Materials

The following supporting information can be downloaded at: https://www.mdpi.com/article/10.3390/vaccines11111708/s1, Table S1: Database Search.
