# Supplementary material for: Systematic Review on the Efficacy, Effectiveness, Safety, and Immunogenicity of Monkeypox Vaccine
Source: Vaccines (Basel). 2023 Nov 10;11(11):1708. doi: 10.3390/vaccines11111708 (PMC10674429; doi:10.3390/vaccines11111708)
Supplement: Supplementary file 1 [file vaccines-11-01708-s001.zip › vaccines-2641450-SI.pdf]

Systematic Review on The Efficacy, Effectiveness, Safety, and Immunogenicity of Monkeypox Vaccine

Ramy Mohamed Ghazy[1] ramy_ghazy@alexu.edu.eg 0000-0001-7611-706X

Ehab Elrewany[1] ehabelrewany@alexu.edu.eg 0000-0002-8700-0630

Assem Gebreal[2] assem.Ahmed1801@alexmed.edu.eg 0000-0002-2155-8500

Rony ElMakhzangy[3] ronyibrahim13@hotmail.com 0000-0002-1089-2677

Noha Fadl[3] nohaosama@alexu.edu.eg 0000-0001-9807-2720

Eman Hassan Elbanna[4] eman.elbanna@alexu.edu.eg 0000-0002-0419-5103

Mahmoud M. Tolba[5] Mahmoud.tolba@alumni2014.guc.edu.eg 0000-0002-6640-3190

Elsayed Mohamed Hammad[2] hmad41337@gmail.com 0000-0002-7523-2346

Naglaa Youssef[6] youssef_naglaa@cu.edu.eg 0000-0002-0368-1759

Hazem Abosheaishaa[7] hazemabosheaishaa@gmail.com 0000-0002-5581-8702

Elsayed Eldeeb Mehana Hamouda[8] elsayedhamouda@gmail.com 0000-0002-7567-8049

Zeyad Elsayed Eldeeb Mehana[2] zeyadhamouda@gmail.com 0009-0006-5673-2018

Ahmed Saad AL Zomia[9] Ahmedszomia@gmail.com 0000-0002-7850-7229

Raad Ahmed A Alnami[9] Araad7768@gmail.com

Emad Ali Saeed Salma[9] Emad2009-1@hotmail.com

Abdulaziz Saleh Alqahtani[9] AbdulazizSalehAQ@gmail.com

Abdulaziz Fayez Alshehri[9] abdulazizalshehri086@gmail.com

Mai Hussein[10,11,12#] mai.mk.hussein@gmail.com 0000-0003-2735-1428

1. High Institute of Public Health, Alexandria University, Egypt
2. Alexandria faculty of medicine, Alexandria university, Egypt
3. Family Health Department, High Institute of Public Health, Alexandria University, Alexandria, Egypt
4. Health Administration and Behavioral Sciences Department, High Institute of Public Health, Alexandria University, Egypt
5. Pharmaceutical Division, Ministry of Health and Population, Faiyum City, Egypt
6. Medical-surgical nursing, Faculty of Nursing, Cairo University, Cairo, Egypt
7. Icahn School of Medicine at Mount Sinai, NY, USA

8. Department of pathology, Faculty of Veterinary Medicine, Alexandria University, Egypt
9. College of Medicine, King Khalid University, KSA
10. Clinical Research Administration, Alexandria Directorate of Health Affairs, Alexandria, Egypt
11. Egyptian Ministry of Health and Population, Cairo, Egypt
12. Master of Medical Science in Clinical Investigation, Harvard Medical School, USA

Running Title: Monkeypox Vaccination: A systematic review

Keywords: Monkeypox; vaccine safety; vaccine immunogenicity; vaccine effectiveness; vaccine efficacy

Corresponding Author: Mai Hussein

mai.mk.hussein@gmail.com ORCID:0000-0003-2735-1428

Alexandria Clinical Research Administration, Health Affairs Directorate, Ministry of Health and Population, Alexandria 21554, Egypt (+201224939670)

Supplementary material

1. **Table S1. Database search**

Data base	Number of citations
**PubMed Medline**	**781**
**PubMed Central**	**460**
**Scopus**	**995**
**WOS**	**713**
**Cochrane Library**	**10**
**ProQuest**	**247**
**European PMC Preprint**	**72**
**MedRxiv**	**32**
**Google Scholar**	**980**
**Total**	**4290**

PubMed Medline (781)

("Monkeypox virus"[MeSH Terms] OR "Monkeypox"[MeSH Terms] OR monkey?pox[All Fields] OR "monkeypox virus*"[All Fields] OR "monkeypoxvirus*"[All Fields] OR "monkey?pox?virus*"[All Fields] OR "Mpox"[All Fields]) AND ("Vaccines"[MeSH Terms] OR "Immunization"[MeSH Terms] OR "vaccin*"[All Fields] OR "immuni*"[All Fields] OR "inocula*"[All Fields]) AND ("Safety"[MeSH Terms] OR "Vaccine Efficacy"[MeSH Terms] OR "Cost-Effectiveness Analysis"[MeSH Terms] OR "immunogenicity, vaccine"[MeSH Terms] OR "adverse effects"[MeSH Subheading] OR "side effect*"[All Fields] OR "Antigenicity"[All Fields] OR "Effectiveness"[All Fields] OR immun*[All Fields] OR "effic*"[All Fields] OR "safet*"[All Fields])

https://pubmed.ncbi.nlm.nih.gov/?term=%28%22Monkeypox%20virus%22%5BMeSH%20Terms%5D%20OR%20%22Monkeypox%22%5BMeSH%20Terms%5D%20OR%20monkey%3Fpox%5BAll%20Fields%5D%20OR%20%22monkeypox%20virus%2A%22%5BAll%20Fields%5D%20OR%20%22monkeypoxvirus%2A%22%5BAll%20Fields%5D%20OR%20%22monkey%3Fpox%3Fvirus%2A%22%5BAll%20Fields%5D%20OR%20%22Mpox%22%5BAll%20Fields%5D%29%20AND%20%28%22Vaccines%22%5BMeSH%20Terms%5D%20OR%20%22Immunization%22%5BMeSH%20Terms%5D%20OR%20%22vaccin%2A%22%5BAll%20Fields%5D%20OR%20%22immuni%2A%22%5BAll%20Fields%5D%20OR%20%22inocula%2A%22%5BAll%20Fields%5D%29%20AND%20%28%22Safety%22%5BMeSH%20Terms%5D%20OR%20%22Vaccine%20Efficacy%22%5BMeSH%20Terms%5D%20OR%20%22Cost-Effectiveness%20Analysis%22%5BMeSH%20Terms%5D%20OR%20%22immunogenicity%2C%20vaccine%22%5BMeSH%20Terms%5D%20OR%20%22adverse%20effects%22%5BMeSH%20Subheading%5D%20OR%20%22side%20effect%2A%22%5BAll%20Fields%5D%20OR%20%22Antigenicity%22%5BAll%20Fields%5D%20OR%20%22Effectiveness%22%5BAll%20Fields%5D%20OR%20immun%2A%5BAll%20Fields%5D%20OR%20%22effic%2A%22%5BAll%20Fields%5D%20OR%20%22safet%2A%22%5BAll%20Fields%5D%29&sort=date&ac=yes

PubMed Central (460)

("Monkeypox virus"[MeSH Terms] OR "Monkeypox"[MeSH Terms] OR monkey?pox[All Fields] OR "monkeypox virus*"[All Fields] OR "monkeypoxvirus*"[All Fields] OR "monkey?pox?virus*"[All Fields] OR "Mpox"[All Fields]) AND ("Vaccines"[MeSH Terms] OR "Immunization"[MeSH Terms] OR "vaccin*"[All Fields] OR "immuni*"[All Fields] OR "inocula*"[All Fields]) AND ("Safety"[MeSH Terms] OR "Vaccine Efficacy"[MeSH Terms] OR "Cost-Effectiveness Analysis"[MeSH Terms] OR "immunogenicity, vaccine"[MeSH Terms] OR "adverse effects"[MeSH Subheading] OR "side effect*"[All Fields] OR "Antigenicity"[All Fields] OR "Effectiveness"[All Fields] OR immun*[All Fields] OR "effic*"[All Fields] OR "safet*"[All Fields])

https://www.ncbi.nlm.nih.gov/pmc/?term=%28%22Monkeypox%20virus%22%5BMeSH%20Terms%5D%20OR%20%22Monkeypox%22%5BMeSH%20Terms%5D%20OR%20monkey%3Fpox%5BAll%20Fields%5D%20OR%20%22monkeypox%20virus%2A%22%5BAll%20Fields%5D%20OR%20%22monkeypoxvirus%2A%22%5BAll%20Fields%5D%20OR%20%22monkey%3Fpox%3Fvirus%2A%22%5BAll%20Fields%5D%20OR%20%22Mpox%22%5BAll%20Fields%5D%29%20AND%20%28%22Vaccines%22%5BMeSH%20Terms%5D%20OR%20%22Immunization%22%5BMeSH%20Terms%5D%20OR%20%22vaccin%2A%22%5BAll%20Fields%5D%20OR%20%22immuni%2A%22%5BAll%20Fields%5D%20OR%20%22inocula%2A%22%5BAll%20Fields%5D%29%20AND%20%28%22Safety%22%5BMeSH%20Terms%5D%20OR%20%22Vaccine%20Efficacy%22%5BMeSH%20Terms%5D%20OR%20%22Cost-Effectiveness%20Analysis%22%5BMeSH%20Terms%5D%20OR%20%22immunogenicity%2C%20vaccine%22%5BMeSH%20Terms%5D%20OR%20%22adverse%20effects%22%5BMeSH%20Subheading%5D%20OR%20%22side%20effect%2A%22%5BAll%20Fields%5D%20OR%20%22Antigenicity%22%5BAll%20Fields%5D%20OR%20%22Effectiveness%22%5BAll%20Fields%5D%20OR%20immun%2A%5BAll%20Fields%5D%20OR%20%22effic%2A%22%5BAll%20Fields%5D%20OR%20%22safet%2A%22%5BAll%20Fields%5D%29

Scopus (995)

(TITLE-ABS-KEY ("Monkeypox virus" OR "Monkeypox" OR "monkey?pox" OR "monkeypox virus*" OR "monkeypoxvirus*" OR "monkey?pox?virus*" OR "Mpox")) AND (TITLE-ABS-KEY ("Vaccines" OR "Immunization" OR "vaccin*" OR "inocula*")) AND (TITLE-ABS-KEY ("Safety" OR "Vaccine Efficacy" OR "Cost-Effectiveness Analysis" OR "immun*" OR "adverse effects" OR "side effect*" OR "Antigenicity" OR "Effectiveness" OR "effic*"))

https://www.scopus.com/results/results.uri?sort=plf-f&src=s&sid=49c60c10bb38d4b69d2c3bd1bed88714&sot=a&sdt=a&sl=404&s=%28TITLE-ABS-KEY%28%22Monkeypox+virus%22+OR+%22Monkeypox%22+OR+%22monkey%3fpox%22+OR+%22monkeypox+virus*%22+OR+%22monkeypoxvirus*%22+OR+%22monkey%3fpox%3fvirus*%22+OR+%22Mpox%22%29%29+AND+%28TITLE-ABS-KEY%28%22Vaccines%22+OR+%22Immunization%22+OR+%22vaccin*%22+OR+%22inocula*%22%29%29+AND+%28TITLE-ABS-KEY%28%22Safety%22+OR+%22Vaccine+Efficacy%22+OR+%22Cost-Effectiveness+Analysis%22+OR+%22immun*%22+OR+%22adverse+effects%22+OR+%22side+effect*%22+OR+%22Antigenicity%22+OR+%22Effectiveness%22+OR+%22effic*%22+%29%29&origin=searchadvanced&editSaveSearch=&txGid=a07a78709a81e6f6572accbe0dc22d55

Web of science (713)

TS=("Monkeypox virus” OR "Monkeypox" OR monkey?pox OR "monkeypox virus*" OR "monkeypoxvirus*" OR "monkey?pox?virus*" OR "Mpox") AND TS=("Vaccines" OR "Immunization" OR "vaccin*" OR "immuni*" OR "inocula*") AND TS=("Safety" OR Efficacy OR "Cost-Effectiveness Analysis" OR "immunogenicity" OR "adverse effects" OR "side effect*" OR "Antigenicity" OR "Effectiveness" OR immun* OR "effic*" OR "safet*")

https://0c10onwc9-1104-y-https-www-webofscience-com.mplbci.ekb.eg/wos/woscc/summary/71cb5e9e-7cb4-4a55-ae10-db865f29107d-8d467dee/relevance/1(overlay:export/end)

Cochrane (10)

"Monkeypox virus" OR "Monkeypox" OR "monkey?pox" OR "monkeypox virus*" OR "monkeypoxvirus*" OR "monkey?pox?virus*" OR "Mpox" in Title Abstract Keyword AND "Vaccines" OR "Immunization" OR "vaccin*" OR "immuni*" OR "inocula*" in Title Abstract Keyword AND "Safety" OR "Vaccine Efficacy" OR "Cost-Effectiveness Analysis" OR "immunogenicity, vaccine" OR "adverse effects" OR "side effect*" OR "Antigenicity" OR "Effectiveness" OR "immunogenicity" OR "effic*" OR "safet*"

Proquest (247)

noft("Monkeypox" OR "monkey?pox" OR "monkeypoxvirus*" OR "monkey?pox?virus*" OR "Mpox") AND noft("Vaccines" OR "Immunization" OR "vaccin*" OR "immuni*" OR "inocula*") AND noft("Safety" OR "Efficacy" OR "Cost-Effectiveness Analysis" OR "immunogenicity" OR "adverse effects" OR "side effect*" OR "Antigenicity" OR "immun*" OR "effec*" OR "safet*")

European PMC Preprint (72)

("Monkeypox" OR "monkey?pox" OR "monkeypoxvirus*" OR "monkey?pox?virus*" OR "Mpox") AND ("Vaccines" OR "Immunization" OR "vaccin*" OR "immuni*" OR "inocula*") AND ("Safety" OR "Efficacy" OR "Cost-Effectiveness Analysis" OR "immunogenicity" OR "adverse effects" OR "side effect*" OR "Antigenicity" OR "Effectiveness" OR "immun*" OR “immunity” OR "effic*" OR "safet*")

MedRxiv Preprint (32)

(Monkeypox OR "Monkey pox") AND (vaccination OR Immunization) AND (Safety OR Effectiveness OR Efficacy OR Immunogenicity) OR "adverse effects" OR "side effect" OR immunity)

Google Scholar (100)

"Monkeypox virus"|"Monkeypox"|"monkey?pox"|"monkey?pox?virus*"|"Mpox" "Vaccines"|"inocula*"|"vaccin*"|"immuni*" "Safety"|"Efficacy"|"Cost-Effectiveness Analysis"|"immune*"|"adverse effects"|"side effect*"|"Antigenicity"|"Effec*"

Total 4290

Endnote Duplicates: 1005

Total new: 3285

2. **Assessment of the study quality**
   https://drive.google.com/drive/folders/1ji4M25ckNTYPitofa3mJVS9xJz324ZJM
